# The Amplified DNA Logic Gates Based on Aptamer–Receptor Recognition for Cell Detection and Bioimaging

**DOI:** 10.3390/bios13110968

**Published:** 2023-11-06

**Authors:** Yajing Wang, Di Wu, Xiuping Cao, Yingshu Guo

**Affiliations:** 1 School of Chemistry and Chemical Engineering, Linyi University, Linyi 276000, China; wyj1468163734@163.com (Y.W.); wudi0279@126.com (D.W.); 15610145928@163.com (X.C.); 2 School of Chemistry and Chemical Engineering, Qilu University of Technology (Shandong Academy of Sciences), Jinan 250353, China

**Keywords:** logic operation, multi-cell analysis, cell receptor, signal amplification, bioimaging

## Abstract

A powerful and accurate method for identifying and isolating cells would be of great importance due to its sensitivity, gentleness and effectiveness. Here, we designed a receptor-based DNA logic device that allows Boolean logic analysis of multiple cells. For ease of expression, the molecules on the cell surface that can bind to the aptamer are referred to as “receptors”. This DNA logic device sends signals based on cell surface sgc8c and sgc4f receptor expression by performing NOT, NOR, AND and OR logic operations, and amplifies and evaluates the signals using HCR. Meanwhile, the release of ICG from the endopore of HMSNs is controlled by affecting structural changes in the DNA logic device. This approach can accurately identify and treat multiple cells on demand based on the presence or absence of cell-specific receptors, facilitating the development of personalized medicine.

## 1. Introduction

Cancers have been one of the diseases with the highest mortality rates, generating a serious threat to human life and health [1,2,3,4]. Early diagnosis of cancer remains the most effective approach [5]. In recent years, DNA nanomaterials have shown great advantages in the field of early disease diagnosis. DNA is addressable and sequence specific because it is assembled tightly according to the complementary base pairing principle [6,7]. Consequently, DNA nanomaterials modified by DNA assembly have improved specificity and selectivity, allowing for the more precise control of a nanomaterial structure and further speciation of nanoprobes for specific diseases. Artificial DNA nanomaterials, such as oligonucleotide-modified nanomaterials, DNA origami and three-dimensional nanomaterials, show promising potential in the field of nanomedicine [8,9,10,11].

The high-precision identification and isolation of living cells using DNA nanotechnology is very challenging. The detection environment is always changing due to the fluid nature of cancer cell membranes in the living environment. So increasing the sensitivity and precision of target molecule detection is essential [12,13]. With the advancement of molecular computer technology, DNA nanomaterials have been developed with logical computing capabilities, which are called DNA logic gate devices. DNA logic gate devices have the characteristics of good biocompatibility, programmability and easy functionalization. Using various endogenous biomolecules as logical inputs, operational strategies for creating cell-level DNA logic can improve the accuracy of molecular recognition. Using it with integrated recognition and analysis capabilities for the logical evaluation of different cells can improve the efficiency of experiments and data analysis and pave the way for accurate diagnosis of diseases [14,15,16,17]. Tan et al. have reported DNA-based logic systems, which can distinguish diseased cells by examining 2 or 3 different expressions of cell markers [18]. At the same time, a dual system for cell subtype specific recognition and siRNA delivery has been reported. This method can not only construct a variety of complex and powerful logic systems, but also open up a new field for cell identification [19,20,21,22,23]. Although logic devices can use different markers as outputs to achieve specific separation of multiple units, low sensitivity caused by weak signals after logic operations is one of the important factors hindering the application of logic devices [18,22,23]. Hybrid chain reaction (HCR) has the characteristics of isothermal experiment, no additional enzymes and high amplification efficiency. It is often used as a signal amplification technique in the field of biosensing [24,25,26]. Here, we intend to design a logic device capable of HCR. It is used to amplify and analyze signals for the presence of receptors on the cell surface. 

Hollow mesoporous silica (HMSNs) are ideal drug delivery vehicles due to their large specific surface area, and improved stability [27,28,29]. In this paper, we want to use carboxyl-modified HMSN as a carrier. At the same time, biological molecules, such as DNA, are linked to the HMSN surface. Due to the different properties of aptamers’ specific recognition of different receptors on the cell surface, logical gate devices were constructed to respond to different cells. According to previous reports, the membrane receptor of Sgc8c aptamers is targeted at tyrosine protein kinase-like 7. Although the cell membrane receptor of Sgc4f aptamer has not been reported, it is clearly indicated in the literature that it is highly selective in binding to cells. Therefore, two aptamer sequences of an Sgc8c aptamer and Sgc4f aptamer will be used in this study [22,23]. 

**Scheme 1:**
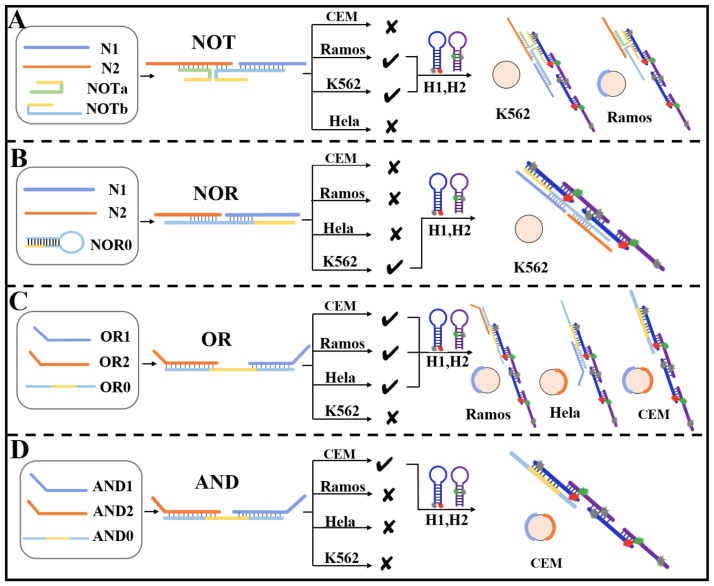
System design and operational mechanism of (**A**) NOT, (**B**) NOR, (**C**) OR and (**D**) AND logic gate.

Specifically, we designed four logic gate devices (NOT, NOR, and, OR) that can respond to different receptors on the cell and output a response signal (Scheme 1). The DNA hybridization simulation diagram of four logic gate devices was shown in Figure S1. These four types of DNA logic gate devices contained two aptamer sequences and one HCR trigger sequence, which can recognize and distinguish cell types based on different receptors on the cell surface.

As illustrated in Scheme 1A, the NOT gate device consists of four parts: NOTa strand, NOTb strand, N1 (containing the sgc4f aptamer strand) and N2 (containing the sgc8c aptamer strand). Specifically, CEM (1,1) possesses both the sgc8c receptor and sgc4f receptor on its membrane surface. These receptors bind specifically to the two aptamer sequences in the NOT gate device, resulting in structural changes. In contrast, K562 (0,0) lacks specific membrane receptors on their surface and does not impact the structure of the NOT gate device. Hela (1,0) only has sgc8c receptors and can solely bind to the sgc8c aptamer. Although the membrane receptor for the sgc4f aptamer was present on the surface of Ramos (0,1), it does not affect the structure of the NOT gate device. The yellow assembly sequences on NOTa and NOTb can hybridize with H1 to trigger the HCR reaction successfully. In the NOT gate device, HCR reaction can be triggered only when the sgc8c receptor is not expressed.

In the present system, H1 and H2 were designed into hairpin structures with fluorophore and quencher. In the presence of trigger chains, H1 (modified Cy5) and H2 (modified FAM) were assembled into long double-stranded DNA, while fluorophore and quencher were separated. So, the fluorescence was restored. In double-stranded DNA, the fluorescence resonance energy transfer occurred between FAM and Cy5. That occurred as follows. FAM was excited by 490 nm light and fluorescence was emitted at ~520nm. Then, Cy5 absorbed the 520 nm light and emitted at ~670 nm fluorescence. This design not only had the advantage of HCR signal amplification, but also reduced background interference.

Scheme 1B shows that the NOR gate device consists of three parts: the NOR0 (containing the trigger strand), N1 (containing the sgc4f aptamer strand) and N2 (containing the sgc8c aptamer strand) strands. If there are sgc8c receptors or sgc4f receptors or both on the surface of the cell, the NOR gate device could be destroyed due to the specific binding of aptamers to receptors. In other words, only when both receptors are not expressed on cell at the same time can the HCR reaction be triggered. In detail, both sgc8c and sgc4f receptors are present on the surface of CEM (1,1), and these two receptors bind specifically to the two aptamer sequences in the NOR gate device. At this time, NOR0 exists in a hairpin structure with 3′-end and 5′-end complementary to each other, and the trigger fragment is hidden. Hela (1,0) and Ramos (0,1) each have one kind of receptors, and can bind specifically to the one kind of aptamer sequence in NOR gate device. So, they cannot expose NOR0 to the trigger sequence either. In general, the three kinds of cells all caused structural alterations in the NOR gate device, resulting in the failure of H1 opening and then the HCR response was not triggered. In contrast, no specific receptors are present on the surface of K562 (0,0). So, the undamaged NOR structure can carry out the later HCR reaction. 

Scheme 1C shows that the OR gate device consists of three parts: the OR0 (containing the trigger strand), OR1 (containing the sgc4f aptamer strand) and OR2 (containing the sgc8c aptamer strand) strand. As long as there are sgc8c receptors or sgc4f receptors or both on the surface of the cell, OR gate device will be destroyed due to the specific binding of aptamers to receptors. This exposes the trigger sequence on OR0 for the subsequent hybridization between the trigger sequence and H1. Specifically, CEM (1,1), Hela (1,0) and Ramos (0,1) can bind to at least one aptamer sequence and all are sufficient at exposing the trigger sequence of the OR0 intermediate site. Then, it continues to incubate with H1 and H2 hairpins for HCR and fluorescent signals are recorded, respectively. Conversely, no specific receptors were present on the surface of K562 (0,0), which had no effect on the structure of the OR gate device. 

Scheme 1D shows that the AND gate device consists of three parts: AND0 (containing the trigger strand), AND1 (containing the sgc4f aptamer strand) and AND2 (containing the sgc8c aptamer strand) strands. Only both sgc8c receptors and sgc4f receptors on the surface of the cell, the AND gate device can be destroyed due to the specific binding of aptamers to receptors. Then, the trigger fragment is exposed. Specifically, two kinds of receptors for sgc8c and sgc4f are all present on the surface of CEM (1,1) simultaneously. So, CEM can destroy the AND gate device, leading to the exposure of the triggering fragment in the intermediate part of AND0. The triggering fragment hybridizes with H1, turning on HCR. Then, fluorescence signals are recorded. Conversely, Hela (1,0), Ramos (0,1) and K562 (0,0) cannot be combined with two kinds of aptamers at the same time. So, the trigger fragment in the middle part of AND0 is not exposed and could not trigger the HCR response. 

## 2. Materials and Methods

### 2.1. Materials

The oligonucleotides (Table S1) and the agarose were obtained from the Sangon Biotech (Shanghai) Co., Ltd. (Shanghai, China). SuperRed/GelRed and cetyltrimethyl ammonium bromide (CTAB) were obtained from Biosharp. 3-triethoxymethilyl propyl succinic anhydride (TESPSA) and Indocyanine green (ICG) were purchased from Macklin Biochemical (Shanghai) Co., Ltd. (Shanghai, China), Tetraethyl orthosilicate (TEOS) was purchased from Condice Chemical (Hubei) Co., Ltd. (Wuhan, China), Ramos cells and Hela cells were purchased from Procell Life Science&Technology Co., Ltd. (Suzhou, China), CEM cells and K562 cells were purchased from iCell Bioscience Inc. (Shanghai, China),The 1640 medium and DMEM medium for cell culture were purchased from Solarbio. The cell counting kit-8 used in the cell experiments was obtained from Dojindo. All animal experimental procedures and techniques were approved by the Animal Ethics Committee of Linyi University, and methods were carried out in ac-cordance with the approved guidelines and laws. 

### 2.2. Apparatus

A Zeta-Size Nano instrument (Zen 3600, Malvern Instruments Ltd., Worcestershire, UK), was used to measure the size of nanomaterials. The morphologies were observed using transmission electron microscopy (TEM) (JEM-2100, Tokyo, Japan), Fluorescence intensity was detected using an F-4600 fluorescence spectrophotometer (Hitachi, Tokyo, Japan), The laser scanning confocal microscopy (LSCM, Nikon C2 Plus) was used to observe fluorescence images.

### 2.3. DNA Logic-Gate-Device Synthesis

#### 2.3.1. Synthesis of “NOT” Logic Gate

N1, N2, NOTa and NOTb sequences were mixed into tubes (200 nM each). The mixtures in tubes were heated at 95 °C and gradually cooled to room temperature over 20 h.

#### 2.3.2. Synthesis of “NOR” Logic Gate

N1, N2 and NOR0 sequences were mixed into tubes (200 nM each). The mixtures in tubes were heated at 95 °C and gradually cooled to room temperature over 20 h.

#### 2.3.3. Synthesis of “AND” Logic Gate

AND1, AND2 and AND0 sequences were mixed into tubes (200 nM each). The mixtures in tubes were heated at 95 °C and gradually cooled to room temperature over 20 h.

#### 2.3.4. Synthesis of “OR” Logic Gate

OR1, OR2 and OR0 sequences were mixed into tubes (200 nM each). The mixtures in tubes were heated at 95 °C and gradually cooled to room temperature over 20 h.

### 2.4. Fluorescence Detection in Solution

A volume of 200 μL NOT/NOR/OR/AND logic gates (200 nM each) were put into tubes. The mixtures in tubes were incubated with 200 μL CEM/Ramos/Hela/K562 cells (10^6^ cells/mL). The mixture was diluted to 1 mL with PBS buffer (pH = 7.4 with 137 mM NaCl and 2.7 mM KCl), respectively. After 30 min, 200 nM H1, H2 were added for 1 h of strand binding and incubation on ice. Next, the fluorescence of the solution was detected. The excitation wavelength was 495 nm, and the fluorescence changes between 500 and 750 nm were recorded. 

### 2.5. cDNA Detection

To test the detection limit of the DNA logic gate, we used the NOT gate as an example. The solutions of 200 μL NOT logic gate was incubated with different concentrations of cDNA for 30 min, respectively. Then, 200 nM of H1, H2 was added for 1 h of chain binding and incubated on ice. Next, the fluorescence of the solution was examined. 

### 2.6. Cell Internalization Experiment

CEM/Ramos/Hela/K562 (1 mL, 1 × 10^5^ cells) were stained with Hoechst 33,342 fluorescent dye in their nucleus, and then incubated with NOT/NOR/OR/AND logic gates for 40 min, respectively. The TCS SP8 II laser confocal microscope was used to observe and cell imaging.

### 2.7. Synthesis of HMSN

Solid silica (sSiO_2_) was synthesized according to the Stöber method. First, 100 mL of ethanol and 4 mL of ammonia were mixed, followed by 3 mL of Tetraethyl orthosilicate (TEOS), which was agitated for 6 h until thoroughly mixed. The surfactant template sol-gel approach was utilized to encapsulate the mesoporous silica shells on sSiO_2_ employing the surface-active agent cetyltrimethyl ammonium bromide (CTAB) as a template to produce the core/shell configuration. The above structures were mixed with CTAB, water, and ethanol for 30 min, and then 1.075 mL TEOS was added to the reaction. After stirring overnight, centrifugation and washing were performed to obtain the sSiO_2_@mSiO_2_ core/shell material. The nanomaterials were dissolved in 0.4 M Na_2_CO_3_ aqueous solution, and the silica nuclei were removed using vigorous stirring. CTAB was removed from the HMSNs using an HCl/ethanol mixture and ultrasound. After that, the HMSNs were obtained by washing the nanomaterials with concentrated HCl/ethanol and water.

For binding to DNA logical devices, 3-triethoxymethilyl propyl succinic anhydride (TESPSA) was used for carboxyl modification on the HMSN surface. When pH = 1, TESPSA can condense with HMSN to form an acid anhydride group. With the increase in pH, the anhydride group is converted to -COOH. Specifically, the synthesized HMSNs were homogeneously dispersed in 50 mL of HCl (0.1 M) and stirred at 50 °C for 5 h after the addition of 0.1 mL of TESPSA. Finally, the carboxy-modified HMSNs (which we denote as HMSN and which we used in all subsequent experiments) was obtained by centrifugation.

### 2.8. Synthesis of HMSNID

A weight of 0.5 mg of the carboxy-modified HMSN was weighed and dissolved in 1 mL of deionized water. An amount of 0.1 mol ICG was added and stirred overnight, and the precipitate was removed by centrifugation. To activate carboxyl groups in HMSN, 200 μL of 0.25 mM EDC and 0.45 mM NHS were added to the precipitate and incubated at 37 °C for 30 min. The supernatant solution was discarded by centrifugation again. Finally, 500 μL of 1.0 μM DNA was added and incubated at 37 °C for 12 h to obtain HMSNID. 

### 2.9. UV Absorption Curves of HMSNs, DNA and HMSN-DNA

The UV absorption curves of 0.005 mg HMSNs, DNA and HMSN-DNA were tested, and their absorption peaks at 260 nm were recorded.

### 2.10. Cytotoxicity Experiments 

CEM/Ramos/Hela/K562 cells were incubated with NOT/NOR/OR/AND logic gates in a cell culture incubator for 40 min, respectively. Next, the cells were irradiated with an 808 nm (1.5 W/cm^2^) laser for 3 min. The cells were cultured for another 6 h and 10 μL CCK-8 was added into each well. After 30 min, cells were detected by a microplate reader. 

### 2.11. The In Vitro Photothermal Performance of HMSNID 

To evaluate the in vitro photothermal performance of HMSNID, CEM/Ramos/Hela/K562 cells were incubated with NOT logic gates for 40 min, respectively. Next, the cells were irradiated with an 808 nm laser (1.5 W/cm^2^) for 4 min. The temperature and corresponding thermal images were recorded.

### 2.12. The In Vivo Experiment 

All animal procedures were approved by the Animal Ethics Committee of Linyi University. Healthy female BALB/c nude mice (5 weeks) were purchased from Vital River Co., Ltd., (Beijing, China). Twenty BALB/c nude mice were randomly divided into four groups (n = 5). Five-week-old Balb/Nude mice were used to inoculate CEM, Ramos, Hela, and K562 (4 × 10^6^ cells/animal), respectively. When the volume of the tumor reached ~150 mm^3^, 100 µL of HMSNID was injected in situ. The mice receiving the same volume of PBS were taken as the control group. During the 21-day period, the body weight of the mice was measured every other day. After 21 days of treatment, the tumors were imaged. 

## 3. Results

### 3.1. In Vitro Multicellular Recognition Assay 

The assembly process of four kinds of DNA logical gate devices using electrophoresis were studied (Figure S2). It is obvious that the results competently prove the successful preparation of the four DNA logic devices.

**Figure 1:**
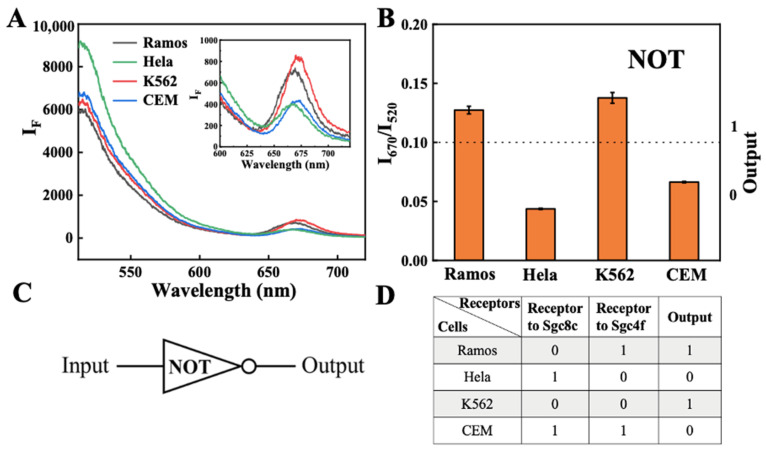
(**A**) Fluorescence intensity of each cell group with NOT gate logic operation function; (**B**) values of fluorescence ratio (I_670_/I_520_); (**C**) logic symbols of NOT gate; and (**D**) truth table corresponding to each cell in NOT gate.

To confirm cell recognition of the NOT gate device, we incubated it with four kinds of cell lines (including Ramos, K562 Hela and CEM), H1 and H2. Then, the resulting fluorescence intensity changes were examined (Figure 1A). At 670 nm, Ramos and K562 had significantly higher fluorescence intensity than Hela and CEM. Based on the experimental data, we established a threshold value of 0.1 (the fluorescence intensity ratio: I_670_/I_520_) for the NOT gate (Figure 1B). When the ratio was above 0.1, the signal output was “1”. When it was less than 0.1, the output was “0”. Figure 1C,D presents the symbol and truth table of the NOT gate device, respectively. It can be seen that if the receptor for Sgc8c (Ramos or K562) was lacking on the cell membrane, the NOT gate device output value was 1. In contrast, cells (Hela or CEM) that can specifically bind Sgc8c had a logic gate output of 0. 

**Figure 2:**
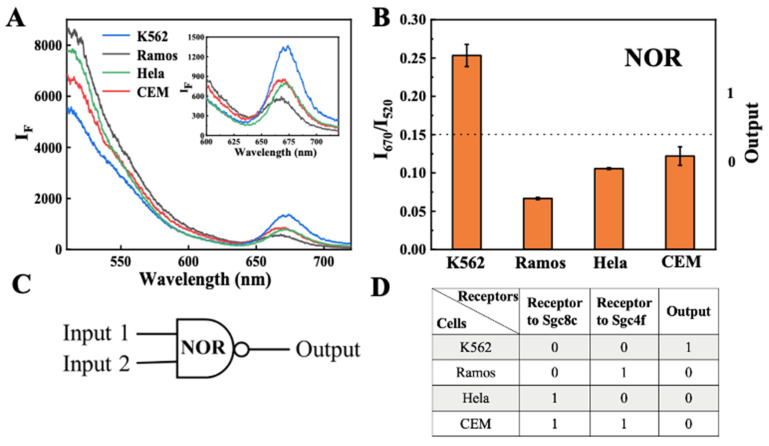
(**A**) Fluorescence intensity of each cell group with NOR gate logic operation function; (**B**) values of fluorescence ratio (I_670_/I_520_); (**C**) logic symbols of NOR gate; and (**D**) truth table corresponding to each cell in NOR gate.

**Figure 3:**
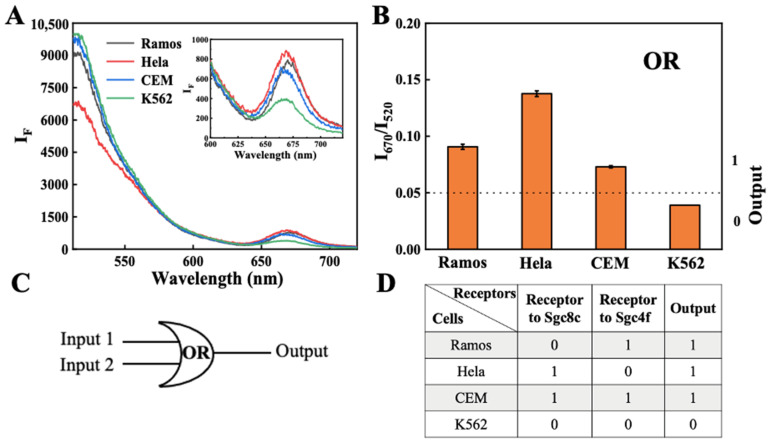
(**A**) Fluorescence intensity of each cell group with OR gate logic operation function; (**B**) values of fluorescence ratio (I_670_/I_520_); (**C**) logic symbols of OR gate; and (**D**) truth table corresponding to each cell in OR gate.

**Figure 4:**
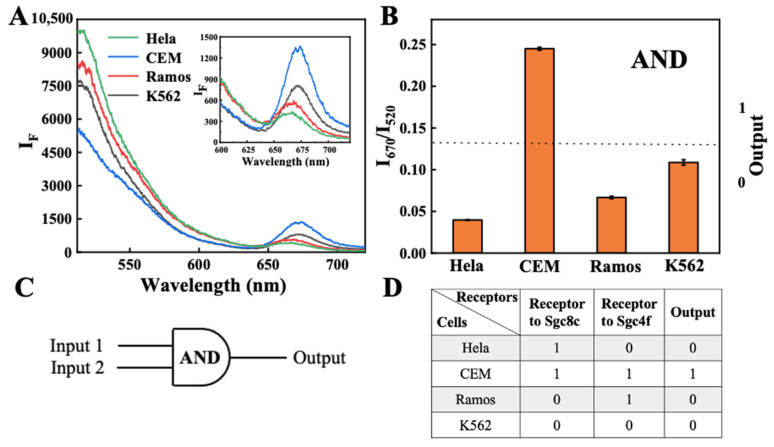
(**A**) Fluorescence intensity of each cell group with AND gate logic operation function; (**B**) values of fluorescence ratio (I_670_/I_520_); (**C**) logic symbols of AND gate; and (**D**) truth table corresponding to each cell in AND gate.

The fluorescence of other logic gates was detected using the same method. And different thresholds were set according to the different ratios within the respective logic gates. The threshold of the NOR logic gate was set to 0.15, the OR logic gate to 0.05 and the AND logic gate to 0.13. As shown in Figure 2, the ratios of K562 were significantly higher than those of the other three cell lines. This was due to the fact that no specific receptor was present on the surface of K562 (0,0). The NOR logic gate sequence structure could not be broken. For the OR logic gate and AND logic gate operations, the ratio difference was due to the length of the intermediate steric acid sequence. The OR gate has a small steric hindrance. The HCR reaction will be opened as long as the receptor is presented. The ratios of CEM, Hela and Ramos were higher than that of K562 (Figure 3). The AND gate has a large steric barrier. Only dual-receptor CEM cells can destroy the AND logic gate device exposing the yellow trigger chain to partially open the HCR reaction. At this moment, the ratios of CEM were higher than those of the other three cell lines (Figure 4).

According to the different logical devices, we obtained signals according to the expression of different receptors on the cell surface. The in vitro multiple on-demand detection and analysis of different cells was achieved by recording and analyzing fluorescence data.

In order to test the sensitivity, the NOT logic gate device was taken as an example. cDNA, which was a complementary strand of N2, acted on behalf of Hela cells. The ratio of fluorescence intensity to different concentrations of cDNA was tested. As shown in Figure S3, the value of I_670_/I_520_ decreased with increasing concentrations of cDNA. A linear relationship was established from 0.01 nM to 1.0 nM with the equation as follows:

I_670_/I_520_ = −0.012 C_DNA_ + 0.091 (R^2^ = 0.99)

in which I_670_/I_520_ represented the ratio of fluorescence intensity 670 nm to fluorescence intensity 520 nm and C_DNA_ represented the concentration of cDNA (nM). The detection limit was estimated to be 3.7 pM based on the 3σ/S calculation (σ is the standard deviation for the blank solution and S is the slope of the linear equation).

### 3.2. Selectivity of Logic Gates

**Figure 5:**
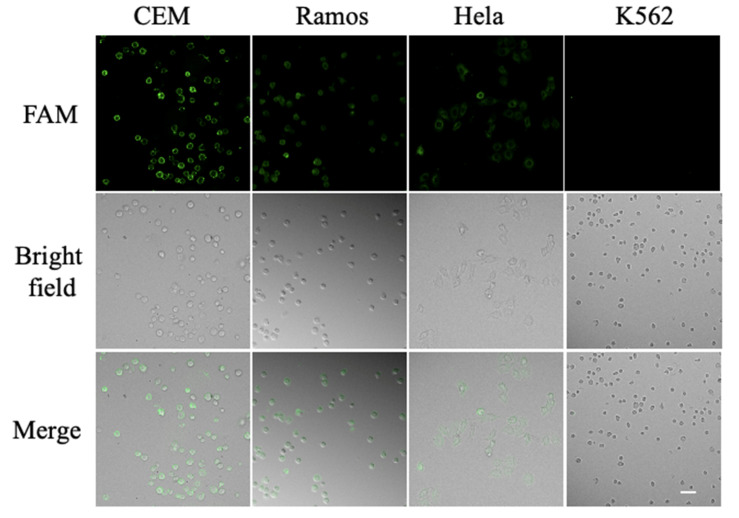
Confocal images of CEM cells, Ramos cells, Hela cells and K562 cells after incubation with NOT’ gate device. Scale bar: 20 μm.

In the four kinds of gate device, the ability of aptamer parts to bind to different cells was investigated. Each cell line showed different fluorescence imaging depending on the types of receptors on the cell membrane. To study the specific binding of aptamers on N1\N2 to cells, N1′ and N2′ (containing FAM) were used to build a new NOT gate device (NOT′ gate device). The 1.0 × 10^5^ Hela, CEM, Ramos, and K562 were cultivated overnight in tiny culture dishes, then treated with the NOT gate device for 40 min, respectively. After centrifugal washing, the products were observed using a laser confocal microscope. From Figure 5, we can see that the fluorescence intensities of Hela and Ramos were weaker than those of CEM and K562. The reasons were as follows. There were two kinds of receptors on the CEM cell surface, which could both specifically bind to N1′ and N2′ at the same time and lead to FAM attaching to the CEM cell surface. Hela or Ramos have only one receptor each. However, there were no receptors on the K562 cells. From the results in Figure 5, it can be concluded that the reported NOT gate device sequences can selectively bind to specific cells. It also illustrated the selective recognition of cells by the NOT gate device. 

In a similar way, the selectivities of the other three logic gates were studied. From Figures S4–S6, we can see the reported three logic gate (NOR′, OR′, AND′ gate device) sequences that can selectively bind to specific cells. That is to say, the other three logic gates (NOR, OR and AND) had similar selectivity.

### 3.3. Nucleic Acid Sequence of NOT Gate Used In Vivo

**Figure 6:**
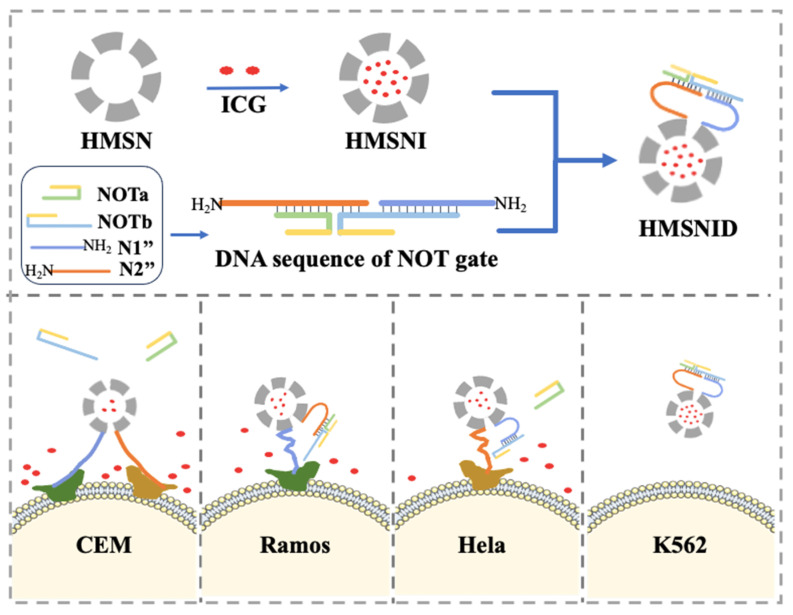
System design and operational mechanism of HMSNID for in vivo treatment.

In order to broaden the application of logic gate research, the NOT gate device as an example was further used in vivo. The system design and operational mechanism are shown in Figure 6. In this part of the research content, the DNA sequence of the NOT gate was used as an example to apply the DNA gate device for therapy in vivo. Amino group-modified N1 and N2 were named as N1′′, N2′′. The N1′′ and N2′′ sequences were attached to carboxy-modified HMSNs through a condensation reaction between the amino and carboxyl. After hybridization among N1′′, N2′′, NOTa and NOTb, the DNA complex structure was good at sealing pores in HMSNs (Figure 6). This moment, ICG was prevented from leakage. Receptors on the surface of cells could control the release of ICG by influencing the DNA complex structure. When the receptor bound specifically to the N1′′ or N2′′, the DNA complex structure was destroyed and ICG was released. The ICG could kill cancer cells through its photothermal effect under 808 nm laser irradiation. Specifically, with regard to the CEM cell, it has two receptors that can bind both sequences (N1′′ and N2′′) from the original structure at the same time. So, the door was completely opened and a lot of ICGs were released. For the Hela cell or Ramos cell, they had only one receptor, which could bind to one sequence (N1′′ or N2′′). There were also some double-stranded nucleic acid structures in the orifice. They more or less affected the outflow of ICG. K562 cells do not have receptor and ICG cannot be released. Therefore, the strategy in Figure 6 could achieve different levels of damage to different cells. It was important to note that the cell types damaged were different from those in Figure 1. We just used the sequence of the NOT gates to study the effect on the viability of different cells types. 

Above all, HMSN and HMSNID (HMSN-ICG-DNA) were synthesized and characterized through TEM analysis (Figure S7A,B) and particle size analysis (Figure S7C,D). In HMSNs we were able to observe around 100 nm with cavities. Next, the assembly process of four kinds of DNA logical gate devices using UV analysis were studied (Figure S8). To test the release of ICG in the presence of cells, HMSNID was incubated with different cells for 40 min, and its mixed solution was examined for relevant temperatures after being irradiated with an 808 nm laser (1.5 W/cm^2^). As shown in Figure S9, CEM cells showed a large temperature increase, Hela cells and Ramos cells showed a weak temperature increase, and K562 cells showed no significant increase in temperature. This warming change is consistent with the receptors contained in various cells. The structure of HMSNID changes to different degrees due to the number of receptors contained in the cells, which leads to a difference in the amount of ICG released and produces a photothermal effect consistent with the number of receptors. The cytotoxic effect of HMSNID were investigated. A number of 1 × 10^4^ cells were treated with HMSNID for 40 min and then irradiated under an 808 nm laser for 3 min. The cells were grown for an additional 6 h. Then, cell viability was analyzed using CCK-8 kits (Figure S10). To distinguish between living and dead cells, the results were further validated using confocal microscopy (Figure S11). The decrease in cell viability was most serious in the CEM group, followed by the Ramos and Hela group, and was negligible in the K562 group. Therefore, it can be concluded that different cells have different degrees of DNA structure stripping on the surface of HMSNID, among which the CEM containing two kinds of receptors had the highest degree of DNA structure stripping. Both types of receptors lacked K562, so the cell viability was almost unaffected.

**Figure 7:**
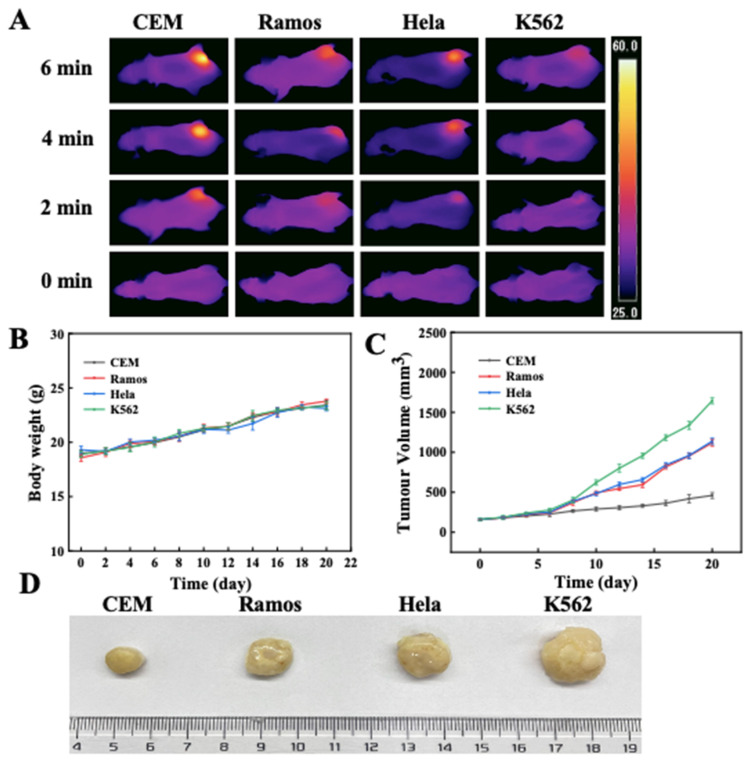
Antitumor effects in vivo of HMSNID. (**A**) Photothermal images of mice tumor with 808 nm laser irradiation. (**B**) Curves of body weight change. (**C**) Curves of tumor volume change. (**D**) Tumor images after 21 days of treatment.

Next, HMSNID was used in vivo to examine its therapeutic effects. Five-week-old Balb/Nude mice were used to inoculate CEM, Ramos, Hela, and K562 (4 × 10^6^ cells/animal), respectively. When the volume of the tumor reached ~150 mm^3^, 100 µL of HMSNID was injected in situ. After 2 h, the area was irradiated with an 808 nm laser. The photothermal effect on the tumor site was evaluated using thermal imaging. As shown in Figure 7A, the temperature rose rapidly in the CEM group, slowly in the Ramos and Hela groups, and barely in the K562 group. These results suggest that HMSNID was expected to play a logical therapeutic role in vivo. So, during the next experiments, the body weight and tumor volume of the mice were recorded (Figure 7B,C). There was no significant difference in the body weight of each group of mice, demonstrating the good biosafety of HMSNID. After 21 days of treatment, the tumors were imaged (Figure 7D). The tumor size in the CEM group was the smallest, suggesting the treatment effect was the most satisfying. The tumor size in the Ramos and Hela groups was slightly larger than that of the CEM group. The tumor size in the K562 group was the largest and the tumor suppression effect was not evident. All these results showed that HMSNID had a good performance for a logical therapeutic in vivo.

Moreover, HMSNID was used in vivo. By examining the experimental data of mice photothermal imaging, tumor volume, mice body weight and H&E staining (Figure S12), they showed an initial good therapeutic effect and biological safety. 

## 4. Conclusions

Sensitive recognition and analysis cells are the cornerstone of improving the efficiency of cancer diagnosis and treatment [25,29]. We designed DNA gate devices (NOT, NOR, AND and OR) by coupling multiple molecular signature inputs into a fluorescence signal. This study enabled the identification and differentiation of cell types based on the different receptors present on the cell surface. Further research, this paper realized the on-demand analysis and simple photothermal therapy of various cancer cell lines by using one of the nucleic acid sequence structures. By substituting different aptamer sequences, this strategy can be widely applied to the logical judgment of other targets, which is helpful for accurate diagnosis. This DNA logic gate devices based on cell surface receptors will also inspire the development of smart nanomaterial applications. 

## Data Availability

All datasets presented in this study are included in the article/Supplementary Material.

## Supplementary Materials

The following supporting information can be downloaded at: https://www.mdpi.com/article/10.3390/bios13110968/s1, Figure S1: schematic diagram of DNA hybridization simulation for four logic gate devices (NOT, NOR, OR, AND); Figure S2: agarose gel electrophoresis results of four DNA logic devices; Figure S3: relationships between the value of I_670_/I_520_ and the concentrations of cDNA (0.01 nM, 0.05 nM, 0.1 nM, 0.5 nM and 1.0 nM); Figures S4–S6: confocal images of CEM cells, Ramos cells, Hela cells and K562 cells after incubation with NOR’/OR’/AND’ gate device; Figure S7: TEM imagines of (A) HMSN and (B) HMSNID. Particle size of (C) HMSN and HMSNID; Figure S8: UV absorption curves of HMSNs, DNA and HMSN-DNA; Figure S9: temperature rise curve of CEM, Ramos, Hela and K562 treated with HMSNID before or after laser irradiation (1.5 W cm^−2^); Figure S10: cell viability of CEM, Ramos, Hela and K562 treated with HMSNID before or after laser irradiation; Figure S11: confocal fluorescence images of CEM, Ramos, Hela and K562 cell viability determined by different treatments. Scale bar = 50 μm; Figure S12: H&E staining of (A) tumor sections and (B) organ sections; Table S1: sequences of oligonucleotides; Table S2: receptor corresponding to Sgc8c aptamers and Sgc4f aptamers in different cell types.
