# Supplementary material for: The Amplified DNA Logic Gates Based on Aptamer–Receptor Recognition for Cell Detection and Bioimaging
Source: Biosensors (Basel). 2023 Nov 6;13(11):968. doi: 10.3390/bios13110968 (PMC10669702; doi:10.3390/bios13110968)
Supplement: Supplementary file 1 [file biosensors-13-00968-s001.zip › biosensors-2599054-supplementary.pdf]

Supplementary Material

The Amplified DNA Logic Gates Based on Aptamer-Receptor Recognition for Cell Detection and Bioimaging

Yajing Wang[1,], Di Wu[1,], Xiuping Cao[1] and Yingshu Guo*[2]

[1] School of Chemistry and Chemical Engineering, Linyi University, Linyi, 276000, China

[2] School of Chemistry and Chemical Engineering, Qilu University of Technology (Shandong Academy of Sciences), Jinan, 250353, China

* Corresponding author.

Email: yingshug@126.com, gyshu@qlu.edu.cn

**NOT**	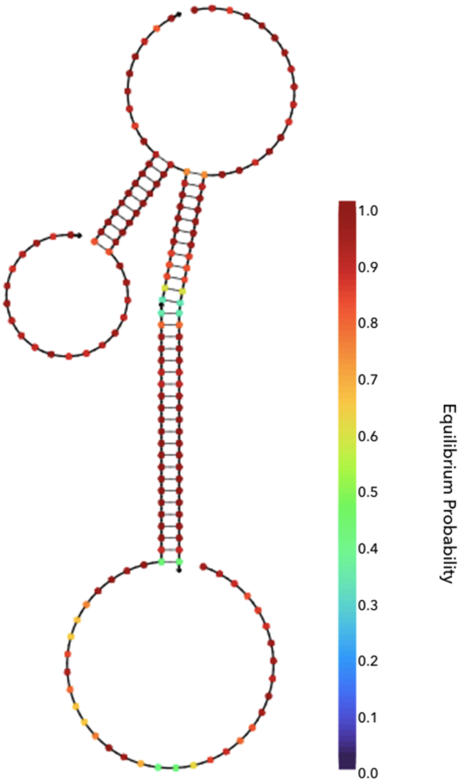 1.0 0.9 0.8 0.7 0.6 0.5 0.4 0.3 0.2 0.1 0.0 Equilibrium Probability	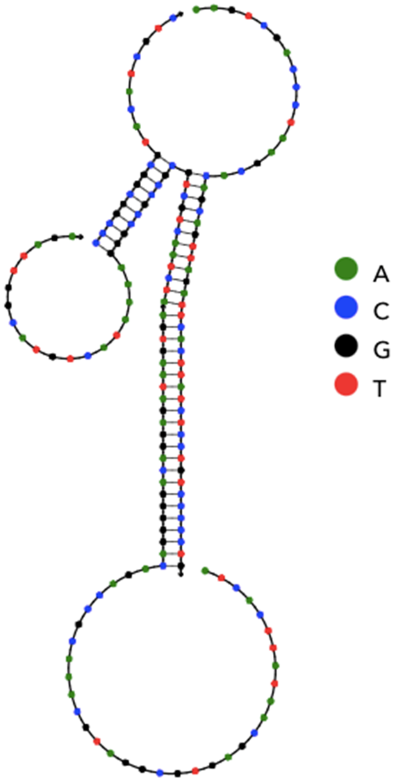 A C G T
**NOR**	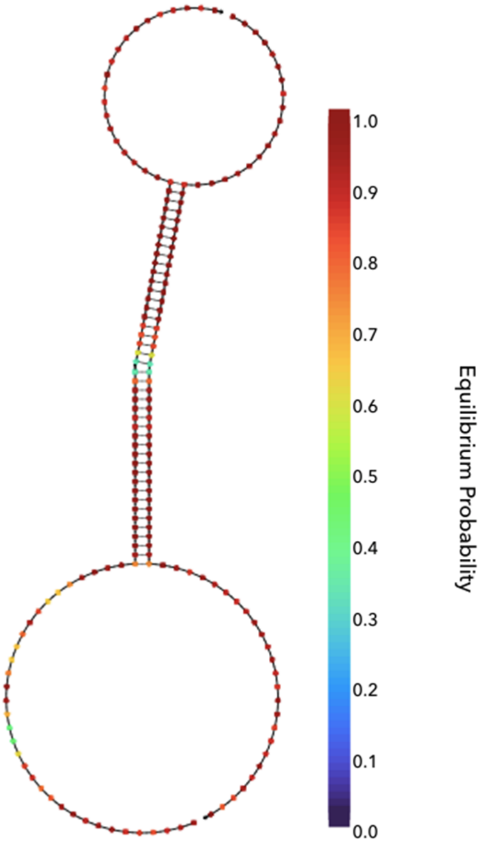 1.0 0.9 0.8 0.7 0.6 0.5 0.4 0.3 0.2 0.1 0.0 Equilibrium Probability	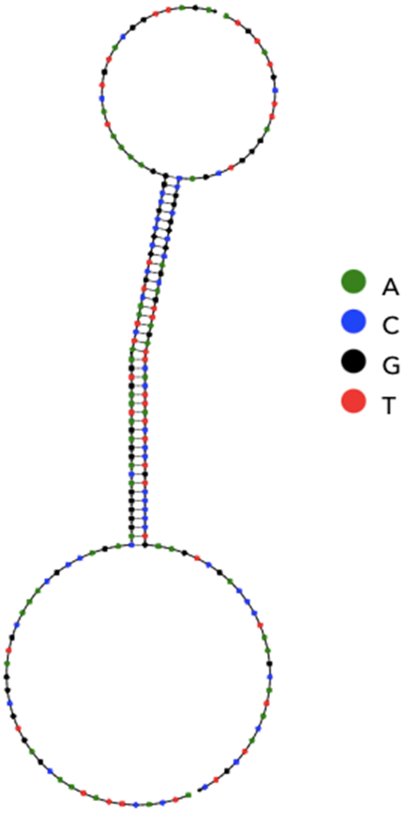 A C G T

**OR**	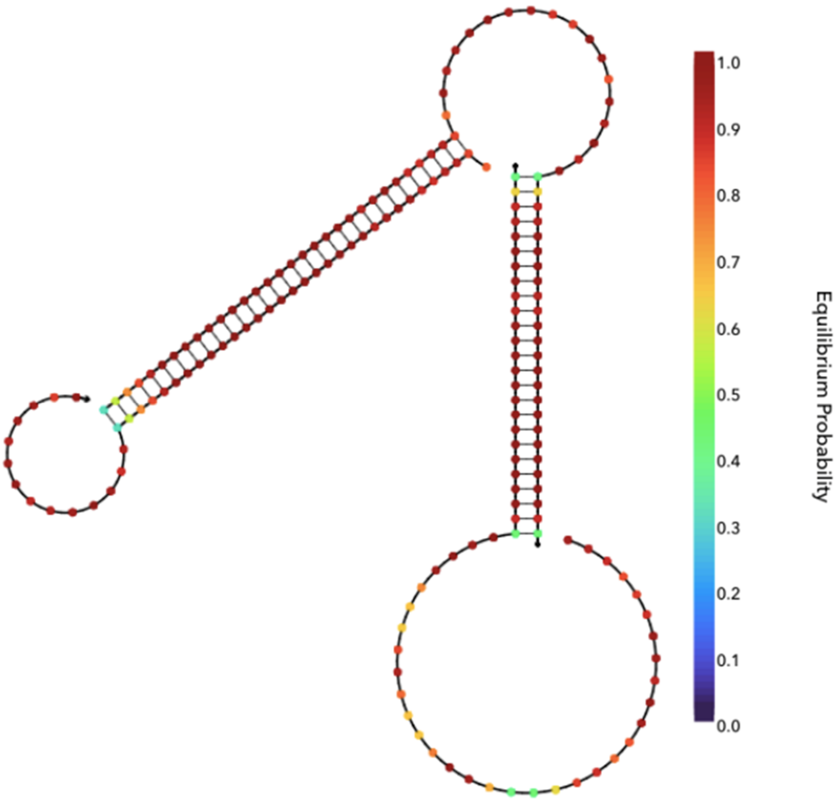 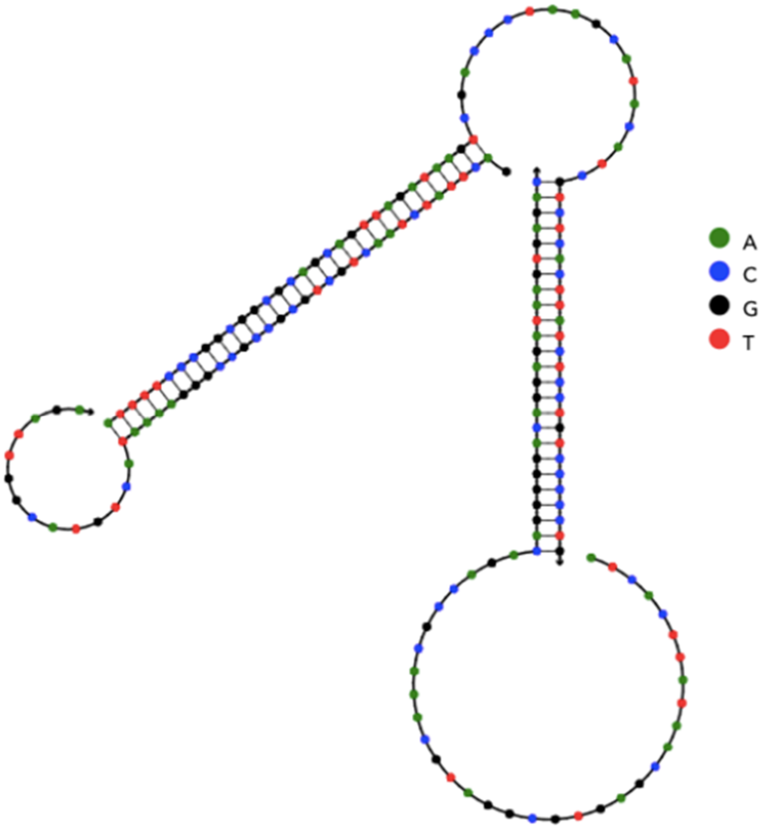

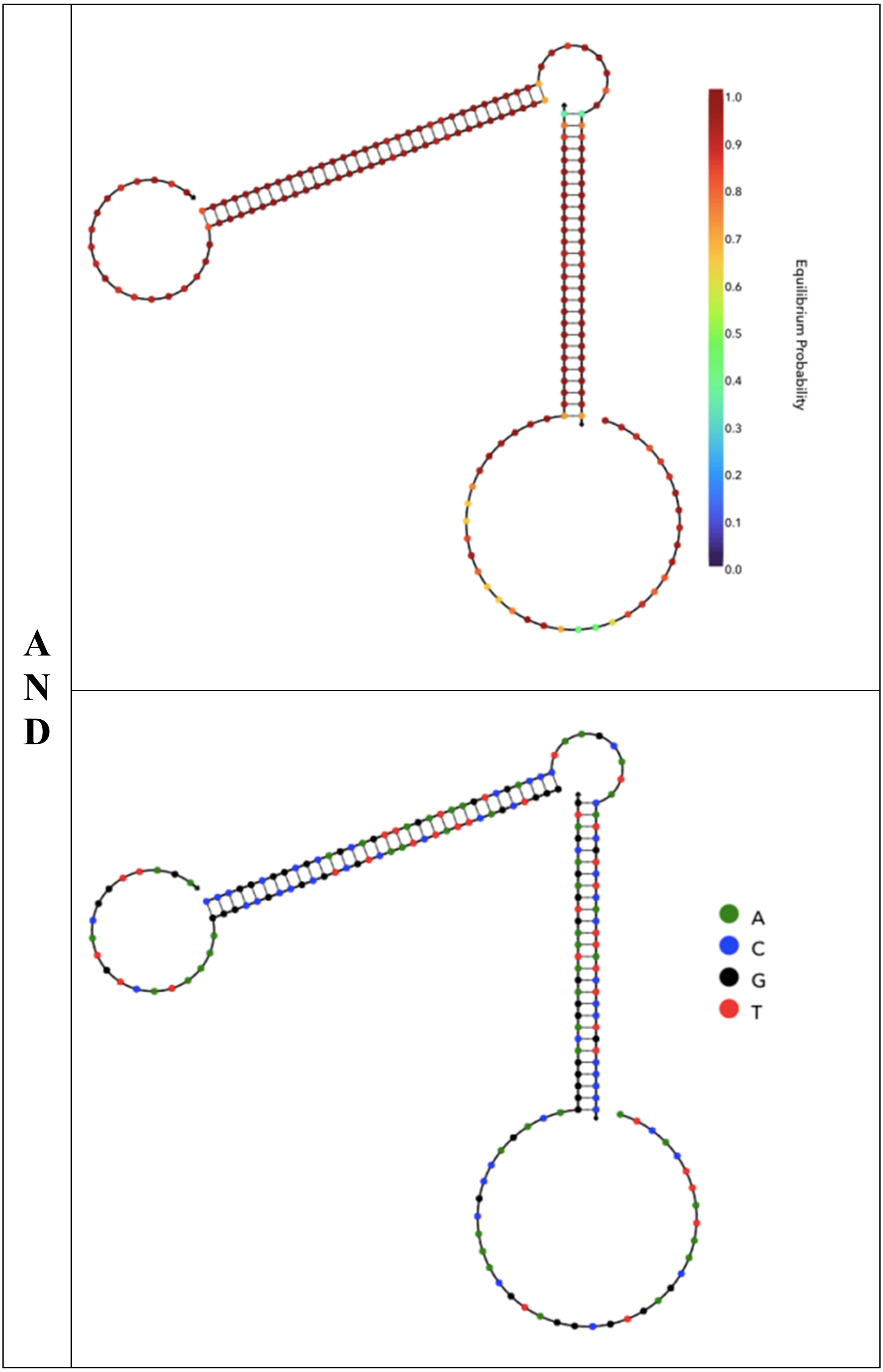

Figure S1 Schematic diagram of DNA hybridization simulation for four logic gate devices (NOT, NOR, OR, AND) from https://nupack.org/.

Characterization of logic gates

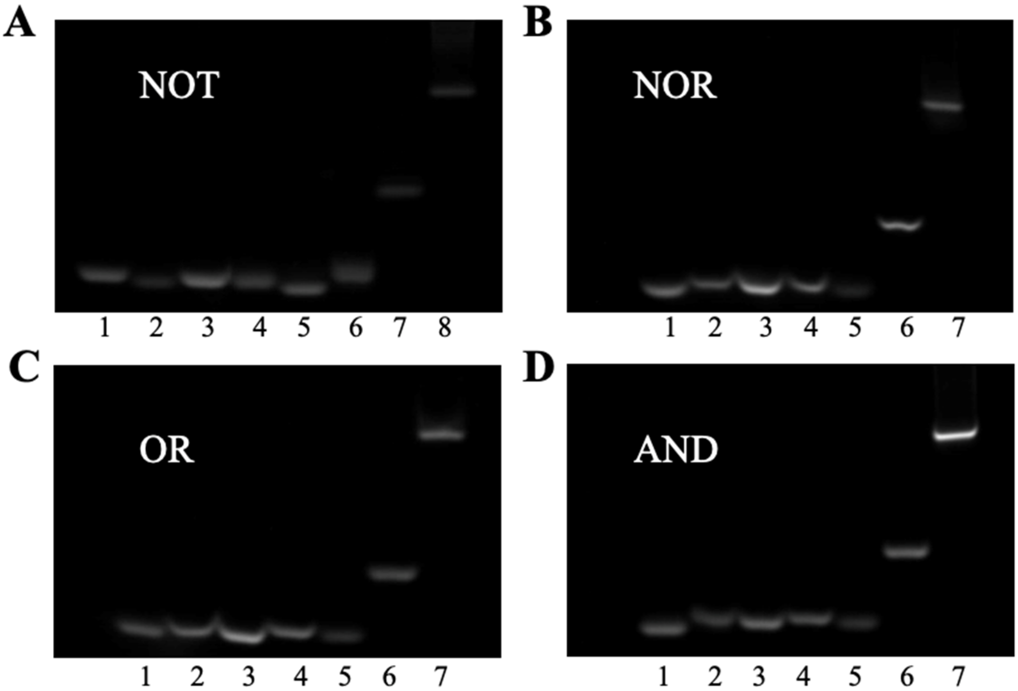

Figure S2 Electrophoretic characterization of four logic gate device (NOT, NOR, OR, AND).

First, agarose electrophoresis analysis was performed for the possibility of four types of gate device (NOT, NOR, OR, AND). The synthesized DNA logic devices were incubated with cells, followed by the addition of H1 and H2. In Figure S2A, the lanes from 1 to 8 were H1, H2, N1, N2, NOTa, NOTb, the product of N1+N2+NOTa+NOTb and the product of N1+N2+NOTa+NOTb+K562+H1+H2. In Figure S2B, the lanes from 1 to 7 were H1, H2, N1, N2, NOR0, the product of N1+N2+NOR0 and the product of N1+N2+ NOR0+ K562+H1+H2. In Figure S2C, the lanes from 1 to 7 were H1, H2, OR1, OR2, OR0, the product of OR1+OR2+OR0 and the product of OR1+OR2+ OR0+Hela+H1+H2. In Figure S2D, the lanes from 1 to 7 were H1, H2, AND1, AND2, AND0, the product of AND1+AND2+AND0 and the product of AND1+AND2+AND0+ CEM+H1+H2. It was obvious that the results of gel electrophoresis not only well prove the successful preparation of the four DNA logic elements, but also prove the subsequent HCR reaction.

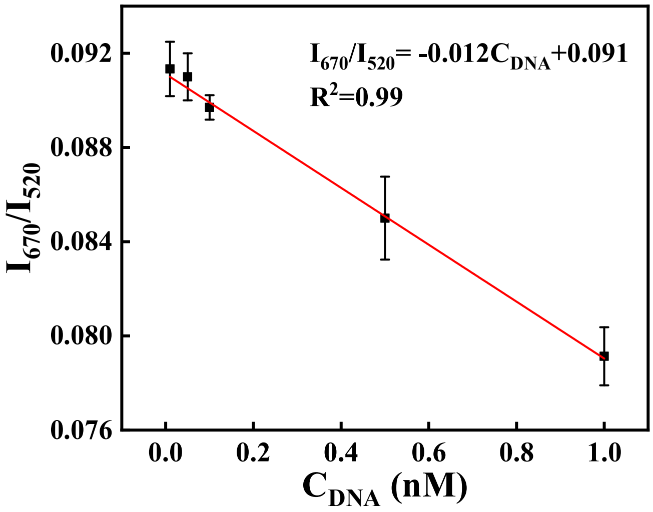

Figure S3 Relationships between the value of I_{670}/I_{520} and the concentrations of cDNA (0.01 nM, 0.05 nM, 0.1 nM, 0.5 nM and 1.0 nM.)

In a similar way, the selectivity of other three logic gates were studied. N1'and N2' (containing FAM) were used to build a new NOR gate device (NOR' gate device). OR1'and OR2' (containing FAM) were used to build a new OR gate device (OR' gate device). AND1'and AND2' (containing FAM) were used to build a new AND gate device (AND' gate device). From Figure S4-S6, the reported three logic gates (NOR', OR', AND' gate device) sequences that can selectively bind to specific cells. The other three logic gates (NOR, OR, AND gate device) had similar selectivity to NOT gate device.

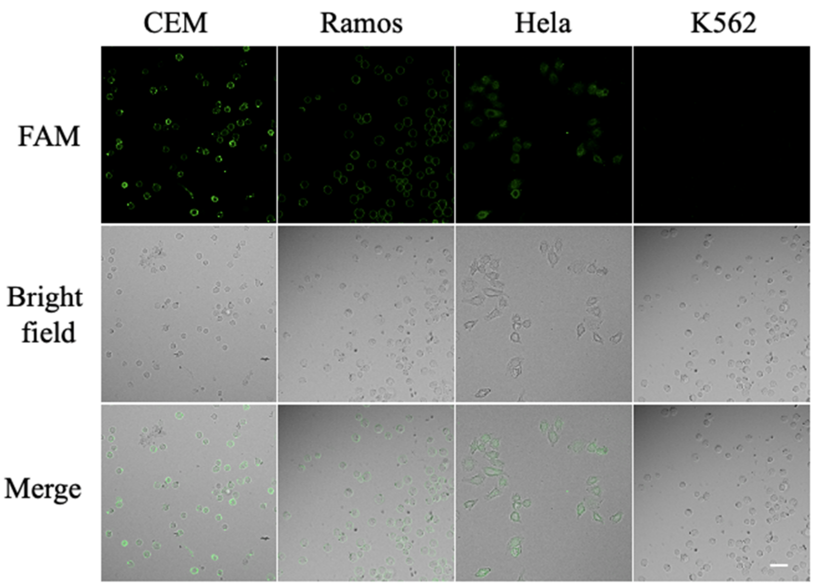

Figure S4 Confocal images of CEM cells, Ramos cells, Hela cells and K562 cells after incubation with NOR' gate device. Scale bar: 20 μm.

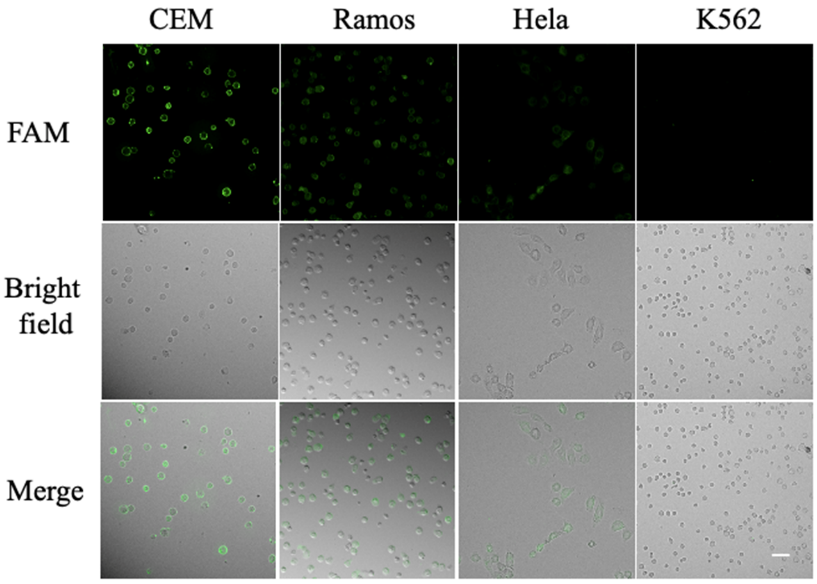

Figure S5 Confocal images of CEM, Ramos, Hela and K562 after incubation with OR' gate device. Scale bar: 20 μm.

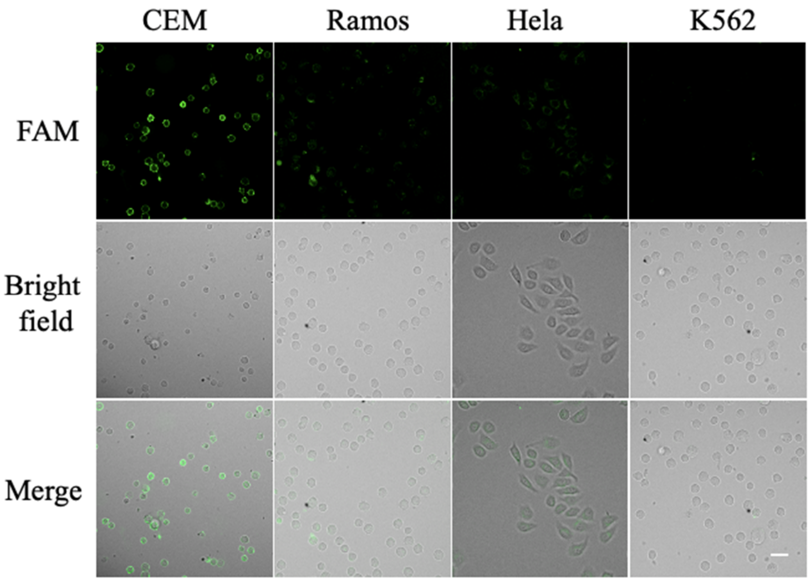

Figure S6 Confocal images of CEM, Ramos, Hela and K562 after incubation with AND' gate device. Scale bar: 20 μm.

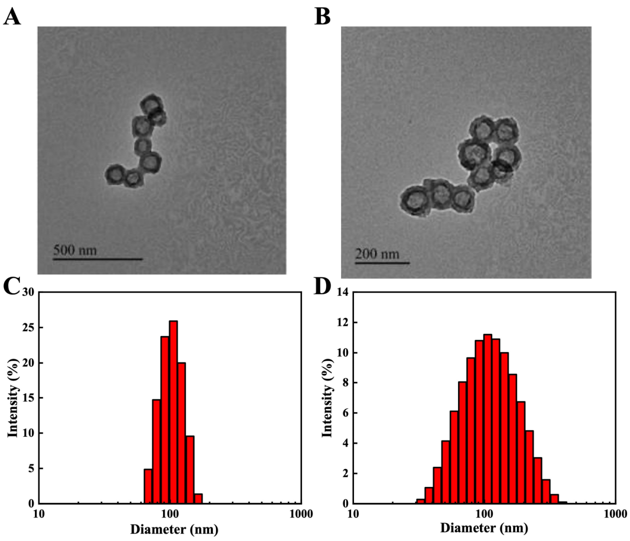

Figure S7 TEM imagines of (A) HMSN and (B) HMSNID. Particle size of (C) HMSN and HMSNID.

Transmission electron microscopy (TEM) and particle size analysis were used for investigating the synthesis of HMSN and HMSNID. From Figure S7A and Figure S7B, HMSN and HMSNID had spherical structure and there were cavities in the middle of the particles. The hydrated particle size of

HMSN was about 100 nm (Figure S7C) and the hydrated particle size of HMSNID slight increased (Figure S7D)

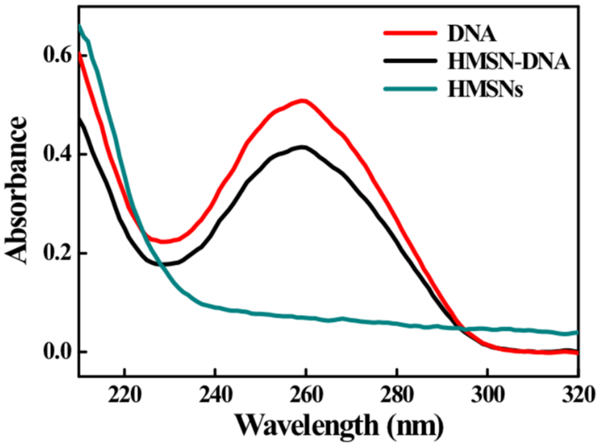

Figure S8 UV absorption curves of HMSNs, DNA and HMSN-DNA.

In addition, through absorption spectrum, HMSN-DNA had absorption peak at 260 nm which corresponded to the DNA absorption peak, demonstrating that DNA was attached on the surface of HMSN (Figure S8).

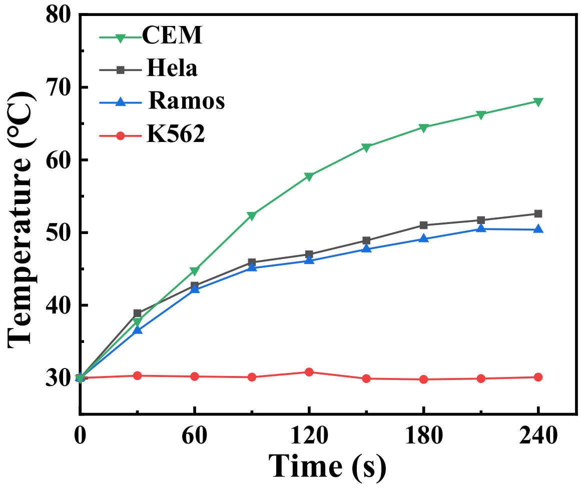

Figure S9 Temperature rise curve of CEM, Ramos, Hela and K562 treated with HMSNID before or after 808 nm laser irradiation (1.5 W cm^{-2}).

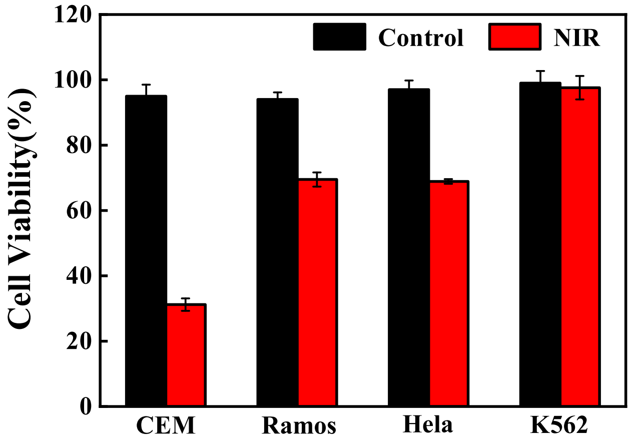

Figure S10 Cell viability of CEM, Ramos, Hela and K562 treated with HMSNID before or after laser irradiation.

Cytotoxic effect of HMSNID was investigated. 1×10^4 cells were treated with HMSNID for 40 min and then irradiated under 808 nm laser (1.5 W/cm^2) for 3 min. The cells were grown for an additional 6 h. Then cell viability was analyzed by CCK-8 kits. The decrease of cell viability was most serious in CEM group, followed by Ramos and Hela group, and was negligible in K562 group (Figure S10). Therefore, it can be concluded that different cells have different degrees of DNA structure stripping on the surface of HMSNID, among which CEM containing two kinds of receptors had the highest degree of DNA structure stripping. All two types of receptors lacked on K562, so the cell viability almost unaffected.

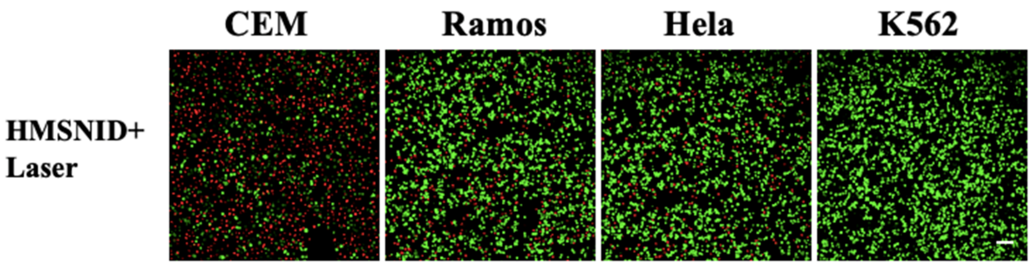

Figure S11 Confocal fluorescence images of CEM, Ramos, Hela and K562 cell viability determined by different treatments. Scale bar = 50 μm.

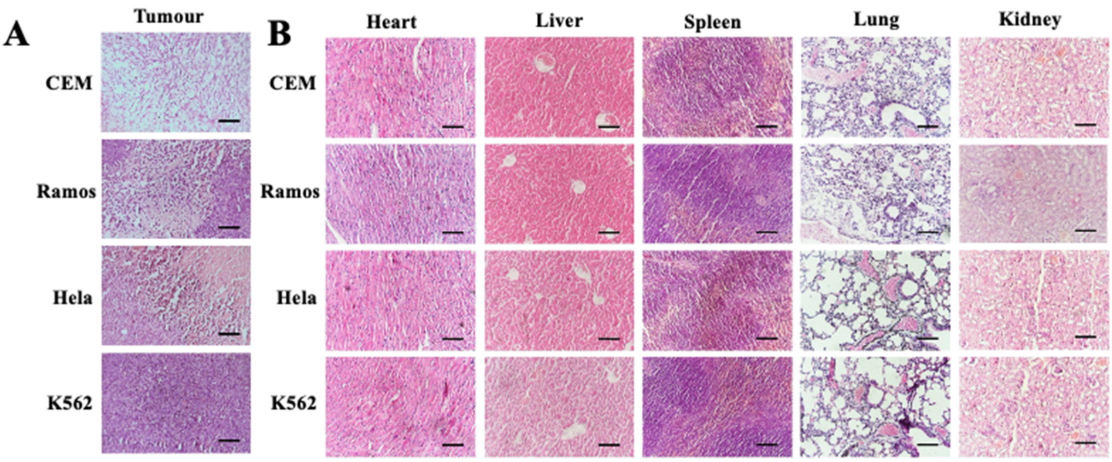

Figure S12 H&E staining of (A) tumor sections and (B) organ sections. Scale bar = 100 μm.

Tumours and major organs (heart, liver, spleen, lung, and kidney) were stained with H&E. As shown in Figure S12A, the tumour cells in the CEM group were extensively necrotic, demonstrating the efficacy of HMSNID as a therapeutic agent. Ramos and Hela tumours exhibited a degree of apoptosis. Compared to the preceding three groups, the K562 group tumour cells proliferated vigorously and had the poorest therapeutic impact, further proving the logical therapeutic efficacy of HMSNID in vivo. In addition, as depicted in Figure S12B, the primary organs (heart, liver, spleen, lung, and kidney) of all mice groups exhibited no substantial abnormalities, which fully demonstrated the good biosafety of HMSNID in vivo and enabled the deployment of logic gates in living creatures.

Table S1. Sequences of oligonucleotides

Logic gate	Name	Sequences(5'-3')
	Sgc4f aptamer	ATC ACT TAT AAC GAG TGC GGA TGC AAA CGC CAG ACA GGG GGA CAG GAG ATA AGT GA
	Sgc8c aptamer	ATC TAA CTG CTG CGC CGC CGG GAA AAT ACT GTA CGG TTA GA
NOT	N1	ATC ACT TAT AAC GAG TGC GGA TGC AAA CGC CAG ACA GGG GGA CAG GAG ATA AGT GA
	N1'	ATC ACT TAT AAC GAG TGC GGA TGC AAA CGC CAG ACA GGG GGA CAG GAG ATA AGT GA-FAM
	N1''	NH_2-ATC ACT TAT AAC GAG TGC GGA TGC AAA CGC CAG ACA GGG GGA CAG GAG ATA AGT GA
	N2	ATC TAA CTG CTG CGC CGC CGG GAA AAT ACT GTA CGG TTA GA
	N2'	FAM-ATC TAA CTG CTG CGC CGC CGG GAA AAT ACT GTA CGG TTA GA
	N2''	ATC TAA CTG CTG CGC CGC CGG GAA AAT ACT GTA CGG TTA GA-NH_2
	cDNA (complementary strand to N2)	TC TAA CCG TAC AGT ATT TTC CCG GCG GCG CAG CAG TTA GAT
	NOTa	CCC GGC GGC GTA CAT CGT C
	NOTb	AAG TCG ACC CTA AGC ACA GCA GTT AGA TTC ACT TAT CTC CTG TCC CCC TG
NOR	N1	ATC ACT TAT AAC GAG TGC GGA TGC AAA CGC CAG ACA GGG GGA CAG GAG ATA AGT GA
	N1'	ATC ACT TAT AAC GAG TGC GGA TGC AAA CGC CAG ACA GGG GGA CAG GAG ATA AGT GA-FAM
	N2	ATC TAA CTG CTG CGC CGC CGG GAA AAT ACT GTA CGG TTA GA
	N2'	FAM-ATC TAA CTG CTG CGC CGC CGG GAA AAT ACT GTA CGG TTA GA
	NOR0	ATG TAT GCT TAG GGT CGA CCG GCG GCG CAG CAG TTA GAT TCA CTT ATC TCC TGT CCC CCT GAA GTC GAC CCT AAG CAT ACA TCG TC
OR	OR1	ATC ACT TAT AAC GAG TGC GGA TGC AAA CGC CAG ACA GGG GGA CAG GAG ATA AGT GAG AC
	OR1'	ATC ACT TAT AAC GAG TGC GGA TGC AAA CGC CAG ACA GGG GGA CAG GAG ATA AGT GAG AC-FAM
	OR2	GAC TTA TCT AAC TGC TGC GCC GCC GGG AAA ATA CTG TAC GGT TAG A
	OR2'	FAM-GAC TTA TCT AAC TGC TGC GCC GCC GGG AAA ATA CTG TAC GGT TAG A
	OR0	ATT TTC CCG GCG GCG CAG CAG TTA GAT AAG TCG ACC CTA AGC ATA CAT CGT CTC ACT TAT CTC CTG TCC CCC TG

AND	AND1	ATC ACT TAT AAC GAG TGC GGA TGC AAA CGC CAG ACA GGG GGA CAG GAG ATA AGT GAG ACG ATG
	AND1'	ATC ACT TAT AAC GAG TGC GGA TGC AAA CGC CAG ACA GGG GGA CAG GAG ATA AGT GAG ACG ATG-FAM
	AND2	GGG TCG ACT TAT CTA ACT GCT GCG CCG CCG GGA AAA TAC TGT ACG GTT AGA
	AND2'	FAM-GGG TCG ACT TAT CTA ACT GCT GCG CCG CCG GGA AAA TAC TGT ACG GTT AGA
	AND0	CCC GGC GGC GCA GCA GTT AGA TAA GTC GAC CCT AAG CAT ACA TCG TCT CAC TTA TCT CCT GTC CCC C
	H1	ATG AAG GAC GAT-BHQ GTA TGC TTA GGG TCG ACT TCC ATA GAC CCT AAG CAT ACA T-Cy5
	H2	GAC CCT-BHQ AAG CAT ACA TCG TCC TTC ATA TGT ATG CT-FAM-TAG GGT CTA TGG AAG TC

Table S2. Receptor corresponding to Sgc8c aptamers and Sgc4f aptamers in different cell types.

Receptors / Cells	Receptor-Sgc8c	Receptor-Sgc4f
CEM	1	1
Ramos	0	1
Hela	1	0
K562	0	0
